# Determinants of Successful Mobile Smoking Cessation Interventions: Cross-Sectional Study

**DOI:** 10.2196/80428

**Published:** 2026-10-27

**Authors:** Bikash K Pradhan, Katerina Lukavska, Roman Gabrhelík, Daniel Novak

**Affiliations:** 1 Department of Cybernetics, Faculty of Electrical Engineering, Czech Technical University in Prague, Technická 2, Praha 6, 16627, Czech Republic, 420 22435 ext 7314, 420 224357314; 2 Department of Analytics, School of Business, Woxsen University, Hyderabad, India; 3 Department of Addictology, Charles University, Prague, Czech Republic

**Keywords:** app-based digital intervention therapy, smoking cessation, influential variables, descriptive statistics, logistic regression, subgroup analysis

## Abstract

**Background:**

In recent years, digital interventions for smoking cessation have seen increasing use, but program completion rates vary widely. Therefore, understanding the factors associated with program completion in cessation programs is critical for evaluating the feasibility and acceptability of the digital intervention program.

**Objective:**

This study aims to identify the predictors (sex, age group, and health) of program completion in a cognitive behavioral therapy–based mobile smoking cessation program.

**Methods:**

Participants enrolled in Adiquit, a mobile app–based smoking cessation program, were classified as completers if they had entered the Withdrawal-Relapse phase and had reported no lapse for 15 consecutive days or had finished all program sessions. User data for this study covered the period from launch through July 2024. Users who did not meet the completion criteria by the end of the study were classified as noncompleters. Predictors were assessed during the Entry and Equilibrium phases, including demographic characteristics, psychosocial factors, and early engagement metrics. Logistic regression was used to identify factors associated with completion.

**Results:**

The three strongest predictors of program completion, as identified by the multivariate model, are higher early engagement (session attendance and screen time), stronger perceived social support, and positive prequitting feelings.

**Conclusions:**

The results of this study will help clarify the variables that lead to successful completion of such a digital intervention program, as well as their importance by sex, age group, health status, and employment status.

## Introduction

Tobacco consumption remains a major global health hazard [1,2]. The smoke in a cigarette contains more than 7000 harmful compounds, causing respiratory, cardiovascular, and cancer diseases [3]. According to the *World Consumption of Cigarettes 1880-2020* report, around 5.2 trillion cigarettes were consumed worldwide in 2020, with China and Europe accounting for the highest consumption [4]. Despite global prevalence programs, reports from the World Health Organization showed only modest declines: 24.4% in 2015, 22.3% in 2020, and a projected 20.4% in 2025 [5]. This has led to a rising concern regarding the inclusion and promotion of various measures for smoking cessation, as well as a need to find an underlying cause to help in decreasing this rate. Mobile-based digital intervention programs have gained prominence due to affordability, accessibility, and independence from direct involvement of health care professionals [6,7]. Rooted in cognitive behavioral therapy (CBT) [8], these apps prepare users for quitting and support abstinence by including gradual behavioral changes [9]. A few examples of such apps include Tobbstop (Universitat Rovira i Virgili) [10], iCanQuit (Fred Hutchinson Cancer Center) [11], QuitGuide (National Cancer Institute) [12], QuitAction (despDev) [13], and SmokeFreeBuddy (23 Ltd) [7]. According to a meta-analysis [14], which included 19 controlled trials, intervention therapy was found to be more effective for smoking cessation than self-help guidelines and no intervention program. While the success rate of quitting without any support is only 5% to 7%, incorporating an app‑based intervention program may increase this rate up to 20% [15].

The process of smoking cessation is complex, and the success of an intervention program depends on multiple interrelated factors, including sociodemographic characteristics, smoking history, underlying health conditions, and financial or employment status [16,17]. Age often shapes these dynamics: older smokers show higher levels of dependence and comorbidities but lower engagement with digital interventions [18]. Sex differences also emerge: women achieve higher cessation rates in community settings [19], whereas men respond better to gamification and engagement metrics [20,21]. Women often use apps for emotional regulation, whereas men use them for instrumental purposes [22]. Self-efficacy strongly predicts success [23], whereas nicotine dependence, hypertension, and cardiovascular disease are associated with quitting but not with completion of the program [24]. Employment status is also important, as occupational stress and workplace policies affect smoking behavior [25,26]. The literature shows inconsistent correlations between these factors and program completion [16,17,27]. The selection of sex, age group, health, and employment status as stratification variables is grounded in three considerations: (1) prior evidence consistently identifies these as moderators of cessation outcomes in both clinical and digital contexts [6,19,26]; (2) each factor is linked to distinct psychological, behavioral, and social mechanisms relevant to quitting—for example, chronic illness may heighten health motivation, while economic status can influence daily routine and stress; and (3) these variables were available for most participants, enabling adequately powered stratified analyses.

The design of digital intervention programs also plays a major role in shaping effectiveness [21]. Zhang et al [21] identified 4 critical factors: external support, personalization, active tracking of user progression, and effective management of withdrawal symptoms [21]. Yet attrition remains high due to poor engagement [28]. Apps that use gamification, rewards, feedback, and frequent interventions reduce dropout rates [6,28]. The digital intervention app Adiquit, used in this study, was designed using established behavioral principles and is therefore expected to be effective in supporting smoking cessation. This study addresses three prespecified research questions: RQ1: Which variables measured within a mobile-based smoking cessation app (Adiquit) are significantly associated with program completion? RQ2: Do the identified predictors of program completion differ across clinically relevant subgroups defined by sex, age group, health status, and employment status? RQ3: Do app-derived behavioral and engagement variables (eg, number of sessions completed, screen time, and social support) demonstrate stronger associations with program completion? Verified abstinence was not included within the scope of this study, and program completion was the primary outcome. The findings were contextualized against a targeted review of comparable app-based cessation studies, with the goal of assessing the generalizability of identified predictors across digital intervention platforms.

## Methods

### Details of the Cessation Intervention Program and App Description

The overall therapy process in the Adiquit app is based on the principles of CBT [29] and is structured as an intensive, multiphase process aimed at guiding people through their smoking cessation journey (Figure 1). The app is designed in accordance with evidence-based principles for digital intervention design, including external support, personalization, active engagement tracking, and withdrawal symptom management. The program starts with the Envisage and Explore (EE) phase, which lasts about 10 sessions and focuses on psychoeducation, the rationale for CBT, motivation evaluation, and personalization of the quit date, with baseline data collected but no social support components. This is followed by the Envisage Quitting (EQ) phase, a critical single session in which the user makes the final decision to quit and identifies their social support network. The Follow-up (FU) phase lasts 21 sessions and focuses on cognitive restructuring, recognizing high-risk situations, increasing self-efficacy, behavioral activation, craving management, and lapse-prevention strategies, which are reinforced through gamified daily check-ins and in-app reminders to engage the support network. Finally, the Withdrawal-Relapse (WR) phase, which spans more than 70 sessions, focuses on abstinence maintenance, relapse prevention, progress tracking, and reward milestones, with features for sharing milestones and keeping participants motivated. As with other CBT-based digital cessation tools [30], its theoretical basis suggests potential utility for smoking cessation; however, no prior independent efficacy studies, especially for Adiquit, are available.

**Figure 1.**
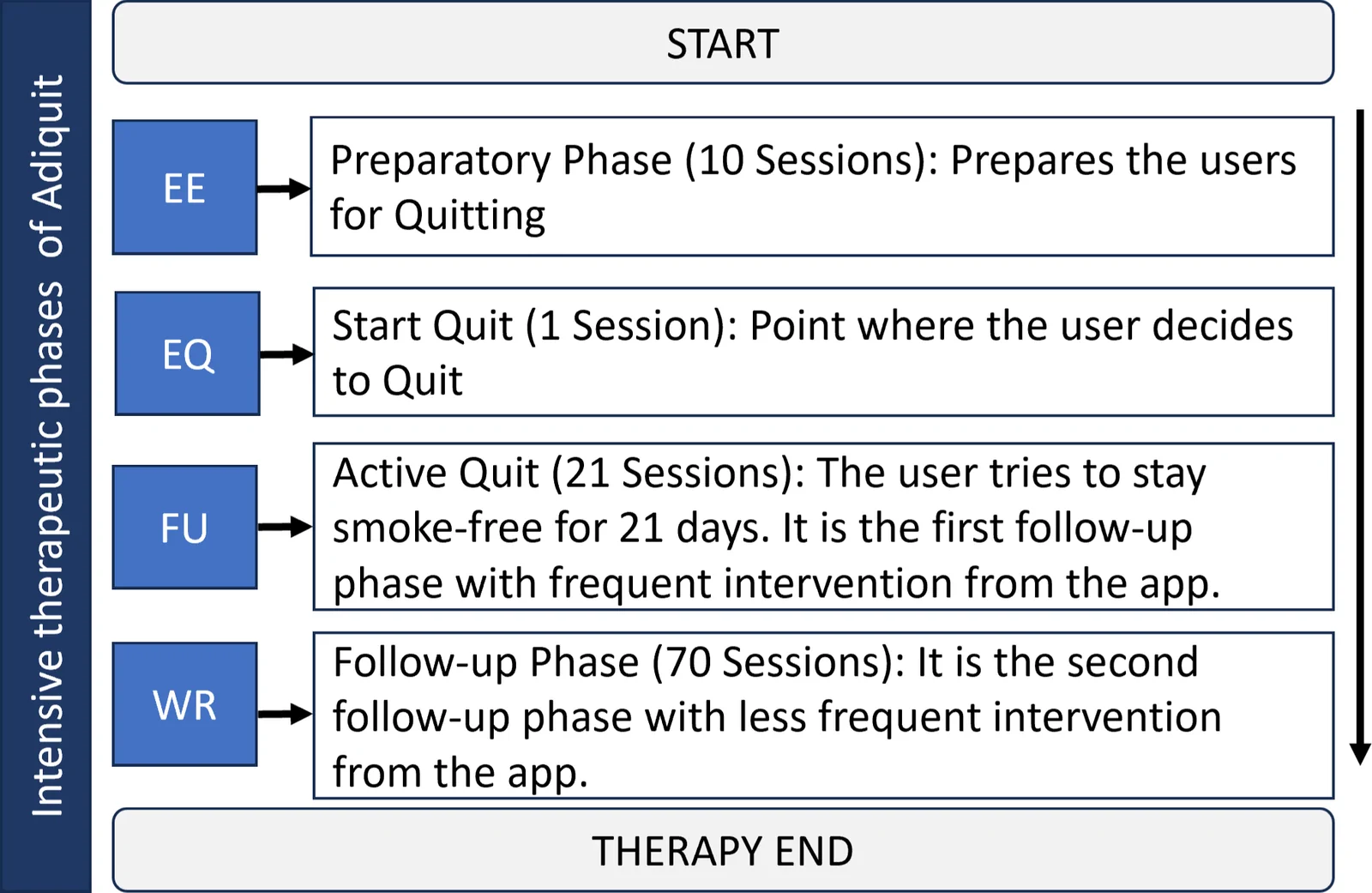
Underlying stages of the cessation intervention therapy in the Adiquit app. EE: Envisage and Explore; EQ: Envisage Quitting; FU: follow-up; WR: Withdrawal Relapse.

### User Information and Data Collection

The app offers a free version that provides access for the first few sessions, and users who opt for a paid subscription (€19.99 per month, where €1=US $1.08, as of July 31, 2024) can access subsequent sessions. Only users who subscribed to the paid tier were included (N=1311 at baseline), as free-tier users were restricted to introductory sessions and lacked exposure to the core intervention components required to assess the study’s primary predictors. According to the available information as of July 2024, 1311 users have opted for the paid service and are considered to be in the initial stage. However, after an initial screening based on the availability of basic user information and on inclusion criteria for the region (the Czech Republic) and age (14‐70 years), only 692 users were included in this study. Ethical approval for the study design and data collection was obtained from the Committee for Ethics in Research of the Scientific Council of the Czech Technical University in Prague. Informed consent for the use of anonymized data for research purposes was obtained electronically from all users during app registration. A detailed description of the approval and consent procedures, privacy safeguards, and compensation is provided in the Ethical Considerations subsection. Basic information, including age, sex, current health conditions, and previous smoking history, was collected at the initial sessions. User responses to several questionnaires (Multimedia Appendix 1) assessing their feelings and perceptions toward the therapy were collected during the therapy, including the preparatory phase. Information on a user’s therapy progress, including sessions and total time invested, was collected from the user log. Individuals who did not complete the programs served as the primary comparator group in this study, but missing outcomes were not included.

### Measures

The information collected from the digital intervention app, including the user’s basic and sociodemographic information, health status, and smoking history, was processed based on whether each variable was binary, categorical, or ordinal. The intervention was delivered via a mobile app and was divided into 4 phases over 4 months, during which the user received a 3- to 10-minute therapeutic dialogue every day covering psychoeducation, CBT-based coping strategies, and withdrawal management. Sessions were asynchronous, with automated reminders sent daily. Personalization was limited to pacing and feedback based on user input. Behavioral information, including the presence of external or social support, the user’s motivation, and feelings toward the therapy, was collected during the initial phases and, in subsequent phases, through the user’s responses to questionnaires. The user’s log data were used to track their involvement in the cessation program by measuring the number of completed sessions and the time spent in the app (screen time). However, only the log information from the first 10 days was considered, as it has been reported that the user disengagement from any intervention program is highest during the first 10 days [31] and is considered an important predictor of program completion. Table 1 presents the list of parameters or measures collected from the app, categorized into 2 groups: digital intervention–based parameters and nondigital intervention–based parameters, including biological, sociodemographic, and information related to previous smoking history and health.

All predictors included in this analysis were measured prior to the onset of the WR phase, which defines the outcome window. Specifically, predictors were derived from the following app phases: (1) sociodemographic, health, and smoking history variables were collected at app registration (EE phase); (2) behavioral questionnaires assessing social support, prequitting feelings, self-efficacy, and motivation were administered during the EQ phase; and (3) log-based engagement metrics were derived from the first 10 days of app usage, which spans the EE and EQ phases. No predictors were measured concurrently with or following the WR phase, precluding reverse causation bias.

**Table 1.** List of data and measures obtained from the app-based digital intervention.

Group and parameter subgroup	Description	Information extracted
Nondigital intervention–based		
Sociodemographic	Includes the basic information of all users	Age, sex, educational status, and town size
User smoking information	Includes the parameters based on the previous smoking history and the type of smoking product used	Age for smoking initiation, cigarettes per day, last nonsmoking period duration, and number of previous quitting attempts
User health information	Includes the parameters with the user’s previous health status and medication history	Health status (healthy or presence of any chronic disease) and medication history (currently on long-term medication or not)
Digital intervention–based		
Behavioral information	Includes all information depicting the user’s motivation and intention toward therapy, either at the initial stage or during the therapy progression	Presence of social support, prequitting feelings, and questionnaires to measure self-efficacy, motivation, and user response to lapse and high-risk situations
Log-based information	Includes the measures derived from the log information	The number of sessions and the screen time during the first 10 days

### Outcome Definition

The primary outcome in this study is “program completion,” operationally defined as (1) completion of the WR phase or (2) active engagement in the WR phase with no self-reported lapse during the preceding 15 days. The outcome criterion was selected for the following reasons: (1) the 15-day lapse-free window aligns with 2-week point prevalence abstinence criteria widely used in cessation trials [32]; (2) entry into the WR phase requires successful completion of preparatory CBT modules, including cognitive restructuring, trigger identification, and quitting date commitment: all of which are theoretically necessary precursors to sustained abstinence [11]; and (3) data from Baumel et al [33] indicate that sustained engagement beyond the first 10 days of digital health intervention is strongly predictive of long-term behavior change outcomes. Here, program completion refers to completing all therapy sessions, whereas verified smoking cessation requires biochemical validation, which was not performed in this study. The proposed outcome in this study reflects persistent behavioral engagement and therapy adherence, which are considered proxies for program completion in digital intervention research [20,34].

### Feasibility and Acceptability

Feasibility was defined as the extent to which users could progress through the program stages, as measured by attrition and completion rates. Acceptability was defined as the extent to which users engaged with the program, as inferred from early session completion (the first 10 days), reported social support, and overall program completion. No direct satisfaction surveys were conducted; therefore, acceptability was assessed indirectly through these engagement indicators.

### Statistical Analysis

#### Descriptive Statistics

After preprocessing, the information collected from the cessation app is split into 2 groups: users who have not finished and users who have finished therapy. Descriptive statistics for the 2 groups were used to understand their distributions. Based on the nature of the collected information (continuous, binary, or ordinal), the choice of the statistical test (*t* test, *χ*^2^ test, or Spearman rank correlation test) was made. Prior to analysis, the completeness of each variable was assessed. Variables with less than 70% response availability were excluded from further analyses (n=4 questionnaire items). For the remaining variables, missing data were handled using complete-case analysis, justified by the observation that missingness was largely attributable to nonprogression through therapy phases (ie, not due to data collection failure), consistent with a missing-at-random assumption.

#### Univariate Logistic Regression

A univariate logistic regression (LR) model was used to assess the individual effect of each predictor on program completion, with each predictor as the independent variable and program completion status as the dependent variable. The results of the univariate analysis are presented in terms of the regression coefficient, SE, *z*-statistic, *P*-value, and odds ratio (OR, 95% CI). A predictor with a *P*<.05 in the univariate analysis was considered significant.

#### Multivariate LR

A multivariate analysis was also performed using all possible combinations of 5 variables from the group of predictors with *P*<.05 in the univariate analysis. Backward stepwise elimination, guided by the Bayesian information criterion, was used to derive the most stable model. Model stability was assessed via 200 bootstrap iterations, with the concordance *C*-statistic and calibration slope reported. Subsequently, the performance of the stratified multivariate model was assessed within subgroups defined by sex (male and female), age group (younger and older), health status (healthy and diseased), and employment status (employed and unemployed) to examine whether the predictors had group-specific effects on program completion.

### Comparison With Previous Literature

A systematic search was conducted in PubMed and Scopus using the keywords: “smoking cessation” OR “Tobacco Cessation” AND (“mobile app” OR “smartphone” OR “digital intervention”) AND (“mobile app” OR “smartphone” OR “digital intervention”) AND (“predictors’’ OR “factors’’), limited to publications from 2020 to 2024. Among the documents obtained, 6 met the inclusion criteria, namely the use of multivariate analysis, app-derived variables, and cessation or program completion outcomes.

### Ethical Considerations

This study followed the Declaration of Helsinki and Czech/European Union (EU) regulations on human research and data protection. Approval was obtained from the Ethics Committee of the Czech Technical University in Prague (approval number: 0000-13/25/51902/EKČVUT, June 2, 2025). Informed consent was obtained electronically during registration in the Adiquit app, with participants informed about the data categories, the voluntary nature of participation, and their withdrawal rights. The app is registered in the Czech Republic, where the legal age of majority is 18; however, adolescent smokers (aged 14‐17 years) were included with parental/guardian consent, consistent with the Czech Research Ethics Board’s guidelines for minor participation in noninterventional digital health research. Only anonymized records of consenting users were analyzed, with identifiers removed and data stored securely on access-controlled servers. Data processing complied with General Data Protection Regulation and Czech law, with no transfers outside the EU [35]. Participants’ rights were respected, and no financial incentives or compensation were provided for participation in this study. The analyzed data were generated as part of users’ voluntary use of the Adiquit app, which they accessed independently via a paid subscription.

## Results

### Descriptive Statistics of the User Data

The descriptive statistics for the data collected from the cessation app for the 2 population groups are presented in Table 2. A total of 692 (333 male and 359 female) individuals were included in this study. Neither male nor female individuals showed a significant difference between the 2 groups (Figure 2). Of all users, only 202 were older than 40 years. The users were divided into 3 categories based on the number of cigarettes smoked per day: light, medium, and heavy smokers. The *P*-value from the *χ*^2^ test was .06, indicating that the difference between the 2 groups is not statistically significant. Other factors, including the duration of the last nonsmoking period, educational background, health status, town size, and employment status, were also compared between the 2 population groups, but no statistically significant differences were observed. However, when compared based on the availability of social support, a significant difference was observed in the *χ*^2^ test (*P*<.001). According to the collected data, 312 users received external support during therapy, whereas 380 did not. Significant differences in the log information between the 2 population groups were observed in each case. The group of users who completed therapy usually had a higher mean number of sessions completed and greater screen time in the first 10 days, with *P*<.001.

**Table 2.** Descriptive statistics of user information collected from the app.

Parameters	Not completed (N=361), n (%)	Completed (N=331), n (%)	*P* value^a^
Age group			.60
Younger (<40 years), (n=490)	252 (69.80)	238 (71.90)	
Older (>40 years), (n=202)	109 (30.19)	93 (28.09)	
Sex			.52
Male (n=333)	169 (46.8)	164 (49.55)	
Female (n=359)	192 (53.18)	167 (50.45)	
Smoking category			.06
Light smoker (n=169)	82 (22.71)	87 (26.28)	
Medium smoker (n=158)	76 (21.05)	82 (24.77)	
Heavy smoker (n=365)	203 (56.23)	162 (48.94)	
Smoking since age (years), mean (SD)	17.5 (5.02)	17.68 (4.73)	.68
Last nonsmoking period duration^b^			.67
<1 week (n=259)	135 (37.39)	124 (37.47)	
1 week-1 month (n=158)	85 (23.54)	73 (22.05)	
1‐3 months (n=115)	59 (16.34)	56 (16.91)	
3‐6 months (n=47)	27 (7.47)	20 (6.04)	
6‐12 months (n=48)	28 (7.75)	20 (6.04)	
>1 year (n=65)	27 (7.47)	38 (11.48)	
Educational background			.53
High school (n=436)	223 (61.77)	213 (64.35)	
University and higher (n=256)	138 (38.223)	118 (35.64)	
Town size			.78
Urban (n=174)	93 (25.76)	81 (24.47)	
Town (n=253)	126 (34.90)	127 (38.36)	
City (n=265)	142 (39.33)	123 (37.16)	
Social support			<.001
Having social support (n=312)	109 (30.19)	203 (61.32)	
No social support (n=380)	252 (69.80)	128 (38.67)	
Health status			.47
Healthy (n=464)	247 (68.42)	217 (65.55)	
Diseased (n=228)	114 (31.57)	114 (34.44)	
Employment status			.23
Unemployed (n=149)	71 (19.66)	78 (23.56)	
Employed (n=543)	290 (80.33)	253 (76.43)	
Adherence value			
Number of sessions in first 10 days, mean (SD)	8.73 (4.38)	12.53 (4.75)	<.001
Screen time in first 10 days (min), mean (SD)	38.35 (26.67)	55.79 (27.21)	<.001
Number of sessions during WT^c^, mean (SD)	16.36 (15.92)	26.35 (16.46)	<.001
Screen time during WT (min), mean (SD)	59.86 (52.22)	89.27 (47.13)	<.001

a *P* values for all parameters except those under “Adherence value” were obtained using the *χ*2 test, and *P* values for variables under “Adherence value” were obtained using the independent *t* test.

b Light smoker: <10 cigarettes/day; medium smoker: 10‐20 cigarettes/day; heavy smoker: >20 cigarettes/day. Urban: <4000 inhabitants; town: ≥4000 inhabitants, but less than that of a city; city: >20,000 inhabitants.

c WT: whole therapy.

**Figure 2.**
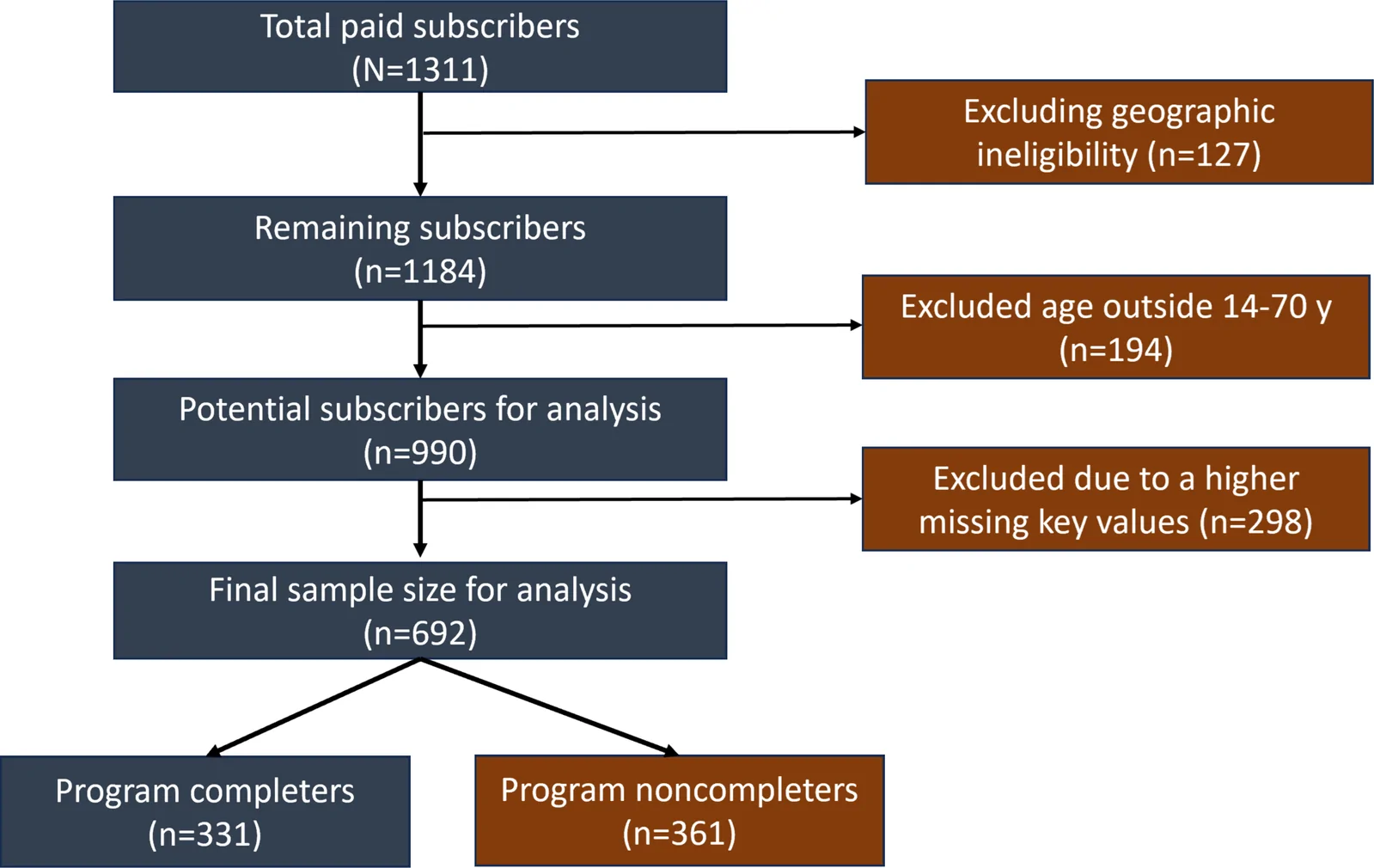
Flow diagram for participant selection.

### Predictors of Program Completion

#### Participation Flow and Attrition

Feasibility was demonstrated by the fact that 662 of 692 registered users progressed to the first engagement stage (EE) and that 187 completed all sessions. However, 331 users, including those who completed all sessions or who were in the WR phase for 15 days without any lag, were considered program completers. Attrition was highest at the EQ stage (173/692, 25%), indicating a critical point where additional support may be needed (Table 3). Acceptability was inferred from engagement metrics: 61.32% (203/331) of users who reported social support completed the program, and higher completion rates were observed among those with greater early session activity. These findings suggest that the intervention was feasible to deliver and acceptable to a subset of users, though direct measures of satisfaction were not collected.

**Table 3.** User attrition rates at different stages of the therapy phase (N=692).

Therapy stage	Users remaining^a^, n	Dropped out before this stage^b^, n (%)
Registered	692	—^c^
Reached EE^d^	662	30 (4.33)
Reached EQ^e^	489	173 (25)
Reached FU^f^	430	59 (8.52)
Reached WR^g^	426	4 (0.57)
Finished	187	239 (34.53)

a Users who reached at least that stage.

b Users whose last recorded activity was in the previous stage (ie, they did not make it this far).

c Not applicable.

d EE: Envisage and Explore.

e EQ: Envisage Quitting.

f FU: Follow-Up.

g WR: Withdrawal Relapse.

#### Univariate Analysis

Univariate LR was used to assess the influence of individual predictors on program completion, and the results are presented in Table 4. Based on the results obtained, social support during therapy and prequitting feelings were among the most influential predictors, with *P*<.001. The participants’ prequitting feelings were assessed during the preparatory phase by asking whether they were afraid of failure during the current cessation attempt. In addition to these parameters, the reason for quitting (eg, job-related) was also found to influence program completion (*P*=.04), with a negative correlation. The log data (number of sessions and screen time) collected over the first 10 days were significant (*P*<.001) and showed a positive correlation with program completion.

**Table 4.** Influence of parameters on program completion.

Parameters and subgroup	Regression coefficient	SE	*z*-statistic	*P* value<|*z*|	Odds ratio (95% CI)
Biological components					
Sociodemographic parameters					
Age	–0.215	0.186	–1.153	.25	0.807 (–0.580 to 0.150)
Age group (younger or older)	–0.159	0.141	–1.125	.26	0.853 (–0.435 to 0.118)
Sex	–0.030	0.110	–0.274	.78	0.970 (–0.245 to 0.185)
Educational background	–0.157	0.125	–1.249	.21	0.855 (–0.402 to 0.089)
Town size	–0.038	0.034	–1.121	.26	0.963 (–0.103 to 0.028)
Employment					
Full-time employee	–0.144	0.092	–1.56	.12	0.866 (–0.324 to 0.037)
Full-time student	–0.204	0.171	–1.19	.23	0.816 (–0.539 to 0.132)
Part-time student	0.337	0.338	0.995	.32	1.400 (–0.326 to 0.999)
Unemployed	–0.375	0.392	–0.957	.34	0.688 (–1.142 to 0.393)
Sick leave	–0.375	0.392	–0.957	.34	0.688 (–1.142 to 0.393)
Part-time job	0.201	0.449	0.446	.66	1.222 (–0.680 to 1.082)
Self-employed	–0.123	0.286	–0.428	.67	0.885 (–0.684 to 0.438)
Parental leave	–0.182	0.428	–0.426	.67	0.833 (–1.022 to 0.657)
Pensioner	–0.182	0.428	–0.426	.67	0.833 (–1.022 to 0.657)
Employment status					
Employed/unemployed	–0.137	0.086	–1.587	.11	0.872 (–0.305 to 0.032)
Smoking history					
Smoking since age regularly	–0.230	0.253	–0.911	.36	0.794 (–0.726 to 0.032)
Cigarettes per day	–0.311	0.189	–1.641	.10	0.733 (–0.681 to 0.060)
Smoking category	–0.052	0.031	–1.657	.09	0.949 (–0.113 to 0.010)
Last quitting attempts count	0.016	0.189	0.085	.93	1.016 (–0.355 to 0.387)
Last nonsmoking period duration	–0.015	0.025	–0.583	.56	0.985 (–0.065 to 0.035)
Nicotine product last used					
PUPW^a^ (cigarette)	–0.127	0.103	–1.23	.22	0.881 (–0.328 to 0.075)
Heated tobacco	–0.134	0.518	–0.258	.79	0.875 (–1.148 to 0.881)
Health and medication status					
Healthy	–0.130	0.093	–1.392	.16	0.879 (–0.312 to 0.053)
Cardiovascular disease	0.693	0.612	1.132	.26	2.000 (–0.507 to 1.893)
Respiratory disease	–0.170	0.261	–0.65	.52	0.844 (–0.682 to 0.342)
Cancer	0.134	0.366	0.365	.72	1.143 (–0.584 to 0.851)
Mental disorders	–0.059	0.243	–0.243	.81	0.943 (–0.534 to 0.417)
History of COVID-19	–0.123	0.128	–0.958	.34	0.885 (–0.374 to 0.128)
Taking regular medication	–0.108	0.093	–1.161	.25	0.898 (–0.291 to 0.074)
App-based intervention components					
Social support					
Having social support	0.622	0.119	5.237	<.001	1.862 (0.389 to 0.855)
Initial feelings about the therapy					
Prequitting feelings	0.263	0.075	3.492	<.001	1.301 (0.115 to 0.411)
Importance of quitting	–0.038	0.021	–1.783	.08	0.963 (–0.079 to 0.004)
Commitment to the quitting plan	0.001	0.026	0.026	.98	1.001 (–0.051 to 0.052)
Last withdrawal method					
Without any help	–0.099	0.097	–1.021	.31	0.905 (–0.290 to 0.091)
Nicotine substitution	–0.127	0.152	–0.836	.40	0.880 (–0.426 to 0.171)
Medication	0.288	0.540	0.533	.59	1.333 (–0.771 to 1.346)
Mobile app	0.085	0.238	0.356	.72	1.088 (–0.381 to 0.550)
Reason for quitting					
Job	–0.799	0.401	–1.989	.04	0.450 (–1.585 to –0.012)
Health	–0.074	0.078	–0.941	.35	0.929 (–0.228 to 0.080)
Social pressure	–0.052	0.086	–0.601	.55	0.950 (–0.220 to 0.117)
Addiction free	0.042	0.109	0.381	.70	1.042 (–0.172 to 0.255)
Finance	–0.024	0.098	–0.244	.81	0.976 (–0.215 to 0.168)
Family	0.037	0.192	0.192	.85	1.038 (–0.340 to 0.414)
Log-based information					
Number of sessions (first 10 days)	0.674	0.187	3.597	<.001	1.962 (0.307 to 1.041)
Screen time (first 10 days), (min)	0.806	0.244	3.298	.001	2.239 (0.327 to 1.285)

a PUPW: product used in past week.

#### Multivariate Analysis

Table 5 presents the results of the multivariate LR model based on 692 participants, demonstrating moderate explanatory power (pseudo-*R*²=0.193) and good discriminative ability (area under the receiver operating characteristic curve [AUROC]=0.772, SD 0.057), though calibration was imperfect (Hosmer-Lemeshow *χ*^2^ statistic [HL χ²_8_]=20.52; *P*=.009). Prequitting feelings (OR 2.447, 95% CI 1.738-3.443), early engagement through the number of sessions completed in the first 10 days (OR 1.158, 95% CI 1.107-1.211), and social support (OR 2.274, 95% CI 1.606-3.218) emerged as strong, highly significant predictors (*P*<.001), consistently retained in bootstrapping. In contrast, screen time during the first 10 days showed no significant effect (OR 1.000; *P*=.10). Collectively, these findings underscore the importance of motivation, early behavioral engagement, and supportive networks as reliable drivers of program success, while passive screen exposure was not a meaningful determinant.

**Table 5.** Result of the multivariate models chosen after using the above method.

Predictors	Odds ratio (95% CI)	*P* value	Bootstrap inclusion (%)
Prequitting feelings	2.447 (1.738-3.443)	<.001^a^	100
Sessions (first 10 days)	1.158 (1.107-1.211)	<.001^a^	100
Social support	2.274 (1.606-3.218)	<.001^a^	99
Screen time (first 10 days), min	1.000 (1.000-1.000)	.10^b^	36

a Statistically significant.

b Not statistically significant.

### Effects of Sex, Age Group, Health, and Employment Status

Table 6 represents the influence of the predictors, stratified by sex, age group, health status, and employment status. The stratified multivariate analysis across 4 demographic dimensions reveals a remarkably consistent pattern: sessions completed within the first 10 days and social support are the most cross-cutting predictors of therapy completion, with significance found in the majority of subgroups regardless of sex, age, or health status. The stratified results indicate that early involvement and psychological preparation are strong, population-level drivers of program completion, whereas demographic factors act as contextual moderators rather than independent predictors.

**Table 6.** Stratified multivariate logistic regression results based on subgroup.

Stratum	Social support		Prequitting feelings		Sessions (first 10 days)	
	OR^a^ (95% CI)	*P* value	OR (95% CI)	*P* value	OR (95% CI)	*P* value
Sex						
Male (n=333)	3.39 (2.00‐5.77)	<.001	2.48 (1.52‐4.06)	<.001	1.20 (1.13‐1.28)	<.001
Female (n=359)	1.74 (1.09‐2.79)	.02	2.51 (1.56‐4.03)	<.001	1.16 (1.10‐1.23)	<.001
Age group						
Younger (<40 y, n=490)	2.95 (1.94‐4.48)	<.001	2.69 (1.78‐4.08)	<.001	1.19 (1.13‐1.25)	<.001
Older (>40 y, n=202)	1.30 (0.68‐2.48)	.42	2.24 (1.23‐4.08)	.008	1.18 (1.10‐1.26)	<.001
Health status						
Healthy (n=464)	3.08 (1.99‐4.76)	<.001	2.10 (1.42‐3.12)	<.001	1.18 (1.12‐1.24)	<.001
Diseased (n=228)	1.33 (0.72‐2.46)	.36	4.80 (2.34‐9.84)	<.001	1.18 (1.10‐1.27)	<.001
Employment status						
Employed (n=543)	2.35 (1.58‐3.50)	<.001	2.34 (1.58‐3.47)	<.001	1.21 (1.15‐1.27)	<.001
Unemployed (n=149)	2.32 (1.08‐4.95)	.03	3.45 (1.73‐6.88)	<.001	1.09 (1.01‐1.18)	.03

a OR: odds ratio.

### Comparison With Previous Literature

Table 7 presents a targeted (nonsystematic) review of studies using app-based interventions for smoking cessation, published between 2020 and 2024, that share comparable design elements (digital interventions, LR or similar analyses, and predictors of app engagement). The comparison reveals consistent evidence that psychological and behavioral variables, as well as sociodemographic characteristics, have a major influence on app-based smoking cessation interventions. Previous research has shown that self-efficacy, motivation, self-affirmation, education, and usability are important predictors of quitting success or program completion. The new study using Adiquit builds on previous findings by including log-based variables, such as screen time and session frequency, as well as contextual factors, such as social support and reasons for quitting (eg, job-related). These findings imply that, in addition to demographics and smoking history, dynamic app engagement and psychological preparation are important factors in influencing cessation outcomes.

**Table 7.** Comparison of the findings of this study with previously reported literature.

Study	Study population (N)	App-based intervention used	Subgroup of variables used	Main findings
Rajani et al [36], 2020	116	Kwit and QuitGenius	Sociodemographic, smoking history, self-efficacy, and motivation	An increase in self-efficacy and motivation from the start to the mid-therapy is positively associated with quitting. No change in the age.
Seaman et al [37], 2021	647	Smoke Free app	Sociodemographic, smoking history, user feelings, and self-affirmation	Users with a greater tendency to continuous self-affirmation are more likely to quit.
Marler et al [34], 2021	391	Pivot program	Sociodemographic, education, income, and baseline intention to quit	Self-efficacy, positive baseline attitude, and higher education contribute to maintaining long-term abstinence.
Borrelli et al [38], 2024	66	Behavioral activation app	Sociodemographic, smoking history, and app satisfaction and engagement	Baseline motivation is an important variable for quitting smoking.
Chen et al [39], 2024	131	quitSTART	Sociodemographic and app usability	Higher app usability positively influences the program completion.
This study	692	Adiquit	Sociodemographic, smoking, and health history, and app-based variables, including psychological, behavioral, and log-based variables	Social support, prequitting feelings, and log parameters (screen time and number of sessions) in the first 10 days, and reason for quitting as job significantly influence the program completion.

## Discussion

### Principal Findings

One of the significant findings from this study’s descriptive statistics is the role of social support in program completion. Social support provides users with a sense of being cared for, loved, and valued, and it also provides a sense of belonging to a community or network [40]. Literature suggests that for humans, social support acts as a stress buffer and has been found effective in improving the success rate of many cessation therapies focused on quitting alcohol, tobacco, opioids, etc [41,42]. As shown in Table 2, 61.32% (203/331) of users with social support completed the Cessation Program, compared with 38.67% (128/331) in the group without social support. Similar findings were observed in the univariate analysis, where social support emerged as a significant predictor (*P*<.001) and positively influenced program completion. A user with social support had 86% (OR 1.862, 95% CI 0.389-0.855) higher odds of completing therapy than a user without social support. The second important finding of the study is the role of a positive perception of therapy completion in the initial days. Information regarding the user’s attitude toward the therapy was collected during the preparatory phase through a prequitting feelings questionnaire (eg, “Are you afraid that you may fail in this therapy?”). The response was collected as either “yes, I am afraid” or “no, I will not fail this time.” Hence, a higher value in the prequitting feelings reflects a positive attitude toward the therapy. In many previous studies, a positive perception of quitting or a positive attitude toward program completion has been associated with better therapy outcomes [43,44]. The results of this study also support this finding and suggest that a positive attitude toward the therapy at the initial stage is associated with a higher likelihood of completing the therapy than a negative attitude. The log information is considered a measure of user engagement in the therapy. According to descriptive statistics and univariate analysis, log-transformed measures (number of sessions completed and total time spent in the app) were positively associated with program completion. The univariate analysis also revealed that users whose primary quitting reason was job showed a lower likelihood of completing the therapy. Gill et al [45] discussed the impact of several reasons for quitting on program completion and investigated the role of intrinsic and extrinsic motivators as reasons for quitting in program completion [45]. They reported that extrinsic factors, such as finances, job satisfaction, and physical appearance, are negatively correlated with program completion. On the contrary, intrinsic factors such as health, well-being, and reduced anxiety increase the likelihood of abstaining and are expected to positively influence cessation outcomes. This explains the negative correlation between the quitting reason (job) and the quitting success, as reported in this study. The study also proposes a stratified multivariate model with bootstrapping and cross-validation, which suggests that social support, prequitting feelings, and number of sessions completed in the first 10 days are important predictors of program completion. Table 6 shows the significance of the obtained predictors in the multivariate analysis across specific subgroups (age, sex, health, and employment status). The stratified multivariate analysis across 4 demographic dimensions reveals a remarkably consistent pattern: sessions completed within the first 10 days and social support are the most cross-cutting predictors of therapy completion, with significance found in the majority of subgroups regardless of sex, age, health, and employment status. However, when the analysis was performed across different groups, some inconsistencies in the findings were observed in the diseased population and the older age group (Table 6), where social support was not significant (*P*=.42). Prequitting feelings are also broadly significant, though with slightly greater fluctuation in smaller strata such as diseased groups, an expected result of lower statistical power rather than a true moderating influence. When age group is considered as a predictor within strata, it contributes inconsistently and rarely approaches significance, supporting the core model’s finding that it has limited independent predictive power. Notably, the older age subgroups have the weakest and least consistent results overall, most likely due to smaller sample sizes and the added complexity of overlapping health and socioeconomic stresses, which may dilute the influence of engagement-based variables. The effect sizes in the healthy stratum, which comprised the majority of participants, closely matched those of the original model, providing confidence in the generalizability of the fundamental findings within these groups. This study has advantages in terms of population size and subgroups of variables considered. Most of the findings presented in this study align with the literature (Table 7), validating and strengthening the efficacy of the intervention method used. However, due to differences in population sizes, study settings, and design methodologies, a direct comparison of the results may be imprecise. The feasibility of the intervention was supported by progression through multiple stages and completion by 27.02% (187/692) of registered users. Acceptability was indirectly evidenced by early engagement and social support, which were associated with higher completion. However, the absence of direct user feedback limits conclusions about perceived acceptability.

### Limitations, Scope for Further Research, and Conclusions

This study focuses on identifying the influential predictors in a digital intervention therapy that helps users quit smoking. Data from 692 users were used to examine the effects of several variables, including sociodemographic, smoking, health history, employment status, and app-derived factors, on program completion. The results suggest that important predictors of program completion in a digital intervention for smoking cessation include social support, prequitting feelings, and the number of sessions completed in the first 10 days. However, these factors may not have a uniform dominant effect when population subgroups are considered by sex, age, health, and employment status. Hence, a population-specific approach is recommended while identifying the influential parameters of program completion in a digital intervention. The study was conducted exclusively among paid subscribers of the Adiquit app, representing a self-selected subgroup of motivated individuals. This limits external validity to the broader population of smokers, particularly those with lower digital health literacy or socioeconomic resources. Future research should examine predictors of cessation engagement across both free and paid user tiers. The retrospective, observational design of this study precludes causal inference. Hence, all identified associations should be interpreted as correlational, and future experimental or longitudinal studies are required to establish causal pathways between the identified predictors and program completion. One major limitation of the study is the lack of clinical validation of users’ quitting. No clinical validation was performed using cotinine levels or exhaled carbon monoxide. Hence, this study is more focused on users’ program completion rather than actual smoking cessation. The presence of potential confounding effects among predictors and population groups was not addressed and should be explored in the future. Feasibility and acceptability were assessed indirectly through attrition and engagement metrics. No direct measures of user satisfaction or qualitative feedback were collected, which restricts the interpretation of acceptability. Future studies should incorporate validated acceptability instruments and user-reported outcomes. In summary, this study highlights the novel role of early engagement metrics and social support in predicting program completion in a digital, CBT-based cessation program delivered via the Adiquit app. Future research should validate these predictors across diverse populations and integrate them into adaptive intervention designs.

## Supplementary material

10.2196/80428 Multimedia Appendix 1 Details of the questionnaires in the therapy stages.

## Abbreviations

AUROC: area under the receiver operating characteristic curve
CBT: cognitive behavioral therapy
EE: Envisage and Explore
EQ: Envisage Quitting
EU: European Union
FU: Follow-Up
HL: Hosmer-Lemeshow
LR: logistic regression
OR: odds ratio
WR: Withdrawal Relapse
