# Supplementary material for: Determinants of Successful Mobile Smoking Cessation Interventions: Cross-Sectional Study
Source: JMIR Form Res. 2026 Oct 27;10:e80428. doi: 10.2196/80428 (PMC13712256; doi:10.2196/80428)
Supplement: Supplementary file 1 [file formative-v10-e80428-s001.docx]

Table S1: Details of the Questionnaires in the Therapy Stages

| **Phase** | **Session / Timing** | **Questionnaire / Information Collected** |
| --- | --- | --- |
| **Phase 1** | Baseline | Age,  sex,  cigarettes/day before therapy,  reason for quitting  “How many cigarettes do you smoke a day?” |
|  | 4 sessions | Why do you want to quit?  How will you feel as a non-smoker?  Importance of life without cigarettes?  How to beat cravings?  When do you feel the urge to smoke?  Strategy for the first day without cigarettes?  Readiness for challenge?  Admit the possibility of slip-ups & coping? - When/where/why does the urge occur?  Time to realise the urge?  First quit attempt?  Fear of failure? |
|  | Ongoing (In case of Lapse) | Why did you start smoking again?  Identify high-risk situations  Plan to avoid them  Confidence in plan?  What could help overcome them? |
|  | Ongoing | Do you have someone to support you?  Will you tell people you are quitting? |
|  | Ongoing | What values/beliefs guide your life?  How has smoking endangered them? |
| **Phase 2** | Pre-Quit or the day of Quit | Confidence in quitting: Why do you believe in yourself? |
| **Phase 3** | Week 1–2 | Quit status (Y/N)  Quit timing (today/tomorrow)  Cigarettes left?  Confidence in staying smoke-free?  Reasons for success/failure?  Coffee consumption (withdrawal):  High-risk situation check,  Feelings after relapse,  Attitude toward smoking at parties  Importance of the goal  Encountered high-risk situations?  Preventing slips (cigarettes on hand?) |
| **Phase 3** | Week 19–23 | Confidence in staying smoke-free for weeks, Motivation & encouragement Partying without smoking, Reviewing reasons for quitting, Causes of slip (8 possible), Confidence in handling high-risk situations, Future imagination (2 years smoke-free), New challenges (smell test, saving money, visiting places), Strategies with other smokers, Sharing success stories, Coping with cravings, Journaling (urges, emotions, pride, high-risk situations), Family smoking history & changes desired, Success celebrations & feedback. |
| **Phase 4** | Week 24–57 | Smoking at work?  Morning routine changes: Healthy habits & emotional triggers, Handling emotions & difficult situations, Breathing exercises, Weekly journaling & challenge feedback, Relaxation methods (3 examples), Stress management (sources & coping), Financial gains from non-smoking (weekly/monthly/yearly), Continued challenges (mindfulness, social support, trips), Reflection on most important past topics. |

*Note: These questions were asked directly to the users, or in some cases, asked to share their experience in certain situations. Some of the questions are mandatory, while others are situational/conditional based on a user's progression through the therapy stages.

**Documents Related to Ethical Clearances:**
